# Contrasting genetic diversity and structure among Malagasy *Ralstonia pseudosolanacearum* phylotype I populations inferred from an optimized Multilocus Variable Number of Tandem Repeat Analysis scheme

**DOI:** 10.1371/journal.pone.0242846

**Published:** 2020-12-08

**Authors:** Hasina Rasoamanana, Santatra Ravelomanantsoa, Noura Yahiaoui, Niry Dianzinga, Emeline Rébert, Miharisoa-Mirana Gauche, Yann Pecrix, Laurent Costet, Adrien Rieux, Philippe Prior, Isabelle Robène, Gilles Cellier, Fabien Guérin, Stéphane Poussier

**Affiliations:** 1 UMR Peuplements Végétaux et Bioagresseurs en Milieu Tropical, Université de La Réunion, Saint-Pierre, Réunion, France; 2 Centre National de la Recherche Appliquée au Développement Rural FOFIFA, Antananarivo, Madagascar; 3 Centre de Coopération Internationale en Recherche Agronomique pour le Développement, UMR Peuplements Végétaux et Bioagresseurs en Milieu Tropical, Saint-Pierre, Réunion, France; 4 Institut National de Recherche pour l’Agriculture, l’Alimentation et l’Environnement, UMR Peuplements Végétaux et Bioagresseurs en Milieu Tropical, Saint-Pierre, Réunion, France; 5 Anses - Plant Health Laboratory - Tropical Pests and Diseases Unit, Saint-Pierre, Réunion, France; Dong-A University, REPUBLIC OF KOREA

## Abstract

The *Ralstonia solanacearum* species complex (RSSC), composed of three species and four phylotypes, are globally distributed soil-borne bacteria with a very broad host range. In 2009, a devastating potato bacterial wilt outbreak was declared in the central highlands of Madagascar, which reduced the production of vegetable crops including potato, eggplant, tomato and pepper. A molecular epidemiology study of Malagasy RSSC strains carried out between 2013 and 2017 identified *R*. *pseudosolanacearum* (phylotypes I and III) and *R*. *solanacearum* (phylotype II). A previously published population biology analysis of phylotypes II and III using two MultiLocus Variable Number of Tandem Repeats Analysis (MLVA) schemes revealed an emergent epidemic phylotype II (sequevar 1) group and endemic phylotype III isolates. We developed an optimized MLVA scheme (RS1-MLVA14) to characterize phylotype I strains in Madagascar to understand their genetic diversity and structure. The collection included isolates from 16 fields of different Solanaceae species sampled in Analamanga and Itasy regions (highlands) in 2013 (123 strains) and in Atsinanana region (lowlands) in 2006 (25 strains). Thirty-one haplotypes were identified, two of them being particularly prevalent: MT007 (30.14%) and MT004 (16.44%) (sequevar 18). Genetic diversity analysis revealed a significant contrasting level of diversity according to elevation and sampling region. More diverse at low altitude than at high altitude, the Malagasy phylotype I isolates were structured in two clusters, probably resulting from different historical introductions. Interestingly, the most prevalent Malagasy phylotype I isolates were genetically distant from regional and worldwide isolates. In this work, we demonstrated that the RS1-MLVA14 scheme can resolve differences from regional to field scales and is thus suited for deciphering the epidemiology of phylotype I populations.

## Introduction

Knowledge of a pathogen’s population biology is a key aspect of disease management [1, 2]. Assessing the genetic diversity and population structure unravels the genetic links between isolates and is useful for developing preventive control strategies [3]. Multiple genotyping techniques have been developed to identify pathogenic isolates, determine phylogenetic relationships between isolates and reveal the genetic links between populations, e.g. for the plant pathogens *Erwinia amylovora* [4] and *Phytophthora ramorum* [5].

MultiLocus Variable Number of Tandem Repeat (VNTR) Analysis (MLVA) is a PCR-based genotyping method for amplifying VNTR loci. VNTR are tandemly repeated DNA sequences, whose copy numbers may vary amongisolates. This variation can be due to two mutational mechanisms during DNA replication: i) the unequal crossing over, frequent among minisatellites with repeat units longer than 9 bp; and ii) the sliding of a strand of DNA followed by a lack of repair ("Slipped Strand Mispairing" or SSM), common for microsatellites with repeat units between 2 and 8 bp [6]. MLVA has the advantage of being highly discriminatory, robust, reproducible and rapid [6, 7]. It has been widely used in medical microbiology [8] to trace the origin of epidemics or the spread of virulent or antibiotic-resistant variants. Later developed for plant pathogenic bacteria, MLVA schemes have proven to be useful for outbreak analysis, epidemiological surveillance and the study of the genetic diversity and population structure of: *Clavibacter michiganensis* subsp. *michiganensis* [9], *Pseudomonas syringae* pv. *tomato* and *maculicola* [10], *Xylella fastidiosa* [11], *Xanthomonas citri* pv. *citri* [12, 13], *Xanthomonas oryzae* [14], *Xanthomonas arboricola* pv. *pruni* [3], *Xanthomonas phaseoli* pv. *manihotis* [15], *Xanthomonas vasicola* pv. *musacearum* [16], *Xanthomonas citri* pv. *viticola* [17], and the *Ralstonia solanacearum* species complex [18–22].

The soil-borne and xylem-limited *Ralstonia solanacearum* species complex (RSSC) causes bacterial wilt (BW) disease on an unusually broad host range, which includes over 50 botanical families [23, 24]. RSSC is one the most devastating plant pathogenic pathogens in terms of the damage it causes to crops [25], such as potato, tomato, tobacco, ginger and banana, as well as leguminous and ornamental crops [24]. BW causes economic losses estimated at US $ 1 billion/year for the potato industry [23]. The RSSC is classified into four major phylogenetic groups (named phylotypes) according to their geographical origin [26] and comprises three species [27, 28]: *R*. *pseudosolanacearum* includes phylotypes I and III originating from Asia and Africa, respectively; *R*. *solanacearum* includes phylotype II with IIA and IIB subdivisions from the Americas; and *R*. *syzygii* includes the phylotype IV from Indonesia, Japan and Australia. Each phylotype is subdivided into unique sequevars based on the partial endoglucanase gene (*egl*) sequencing [29].

In Madagascar, BW disease was first reported in 1934 by Bouriquet on tobacco [30] and artichoke [31]. Later, BW was described on potato, eggplant, groundnut, tomato [32], bean and cabbage [33]. BW was well established in the central highlands, as well as in western and eastern lowlands, where a few RSSC isolates were collected and identified as belonging to phylotypes I and III [34] (Philippe Prior, 2006, unpublished work,). In 2009, major outbreaks were reported in the main potato production areas of the central highlands [19]: BW, which usually occurs on rain-fed potato crops during the rainy season, appeared in the winter season on irrigated potato crops previously unaffected by the disease [19, 35]. In addition, BW symptoms appeared very early during vegetative growth and the BW-tolerant cultivars, developed by the National Centre for Rural Development and Applied Research (FIFAMANOR) and distributed to farmers, became susceptible to RSSC [19].

In 2013, a wide sampling campaign was conducted in Madagascar’s central highlands. A total of 1224 isolates were collected: 10% belonged to phylotype I, 72% to phylotype IIB-1 and 18% to phylotype III [19]. Only the population genetics of phylotypes IIB-1 and III were analysed, using the MLVA schemes RS2-MLVA9 for phylotype II isolates and RS3-MLVA16 for phylotype III isolates [19], derived from previously published studies [20, 21]. This study revealed contrasted population structures between phylotype IIB-1 and phylotype III. Findings suggest that phylotype IIB-1 isolates, reported in Madagascar for the first time, were introduced and spread massively via latently infected potato seed tubers, whereas Malagasy phylotype III isolates appeared to be endemic [19].

The present study aims to develop and apply an optimized MLVA scheme specifically designed for phylotype I isolates in order to: (i) assess the level of genetic diversity and structure of phylotype I populations isolated in Madagascar’s central highlands and lowlands, (ii) compare the population genetic structures of phylotype I with Malagasy phylotypes IIB-1 and III isolates and (iii) analyse the potential genetic links of the Malagasy phylotype I isolates with isolates from other countries.

## Materials and methods

### Bacterial isolates and phylogenetic assignment

Two collections of Malagasy RSSC isolates belonging to phylotype I and maintained at the Plant Protection Platform (Saint-Pierre, Réunion, France) were used in this study. The first collection included 123 isolates from Analamanga (n = 28) and Itasy (n = 95), regions in the central highlands (mean elevation = 1057.23 m). The second collection included 25 isolates from the Atsinanana region in the lowlands (mean elevation = 28.31 m) (S1 Table). The regional difference in elevation provided the opportunity to study the influence of altitude on the genetic diversity and structure of phylotype I populations. In addition, the Analamanga and Itasy regions are in the main vegetable producing areas in Madagascar, where a devastating BW outbreak occurred in 2013. In the Atsinanana region, several cases of BW were also reported. Madagascar’s main seaport is in this region (Toamasina), which allowed the study of the potential influence of frequent commercial exchanges of plant material on the genetic diversity of phylotype I isolates.

**Fig 1:**
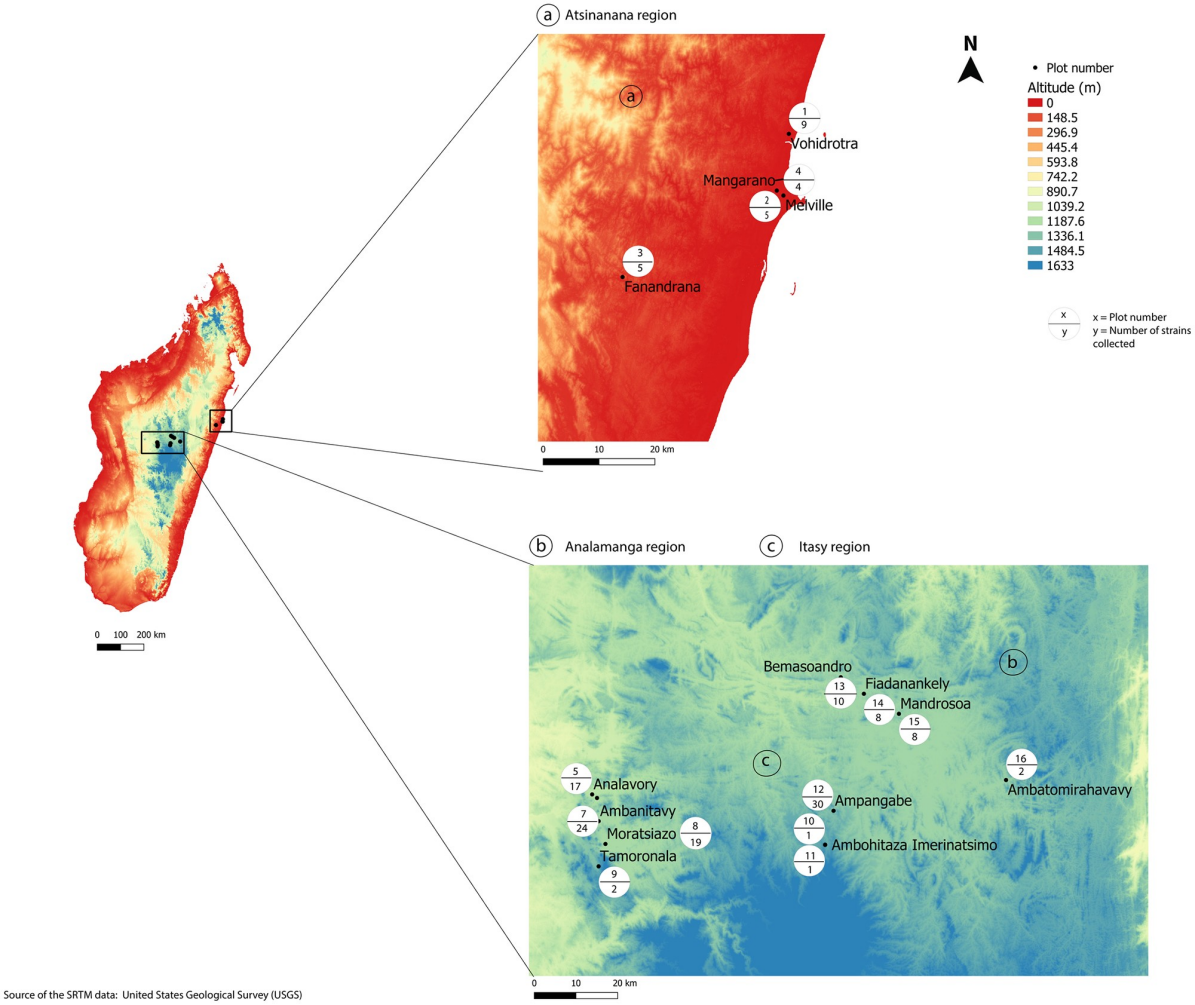
Collection area of RSSC phylotype I in Madagascar. The 16 sites where phylotype I isolates were collected in the three Malagasy regions (a: Atsinanana; b: Analamanga; and c: Itasy) are indicated by black dots. Each white circle contains information about the plot number and the number of isolates collected in each site.

The 148 Malagasy isolates were collected from stem fragments of seven plant species (*Solanum lycopersicum*, *S*. *tuberosum*, *S*. *melongena*, *S*. *aethiopicum*, *S*. *nigrum*, *S*. *scabrum*, *Capsicum annuum*) collected from 16 fields (Fig 1) in the Atsinanana region (4 plots), the Analamanga region (4 plots) and the Itasy region (8 plots). All the Malagasy isolates were checked by multiplex PCR [29] in order to confirm their assignment to RSSC and phylotype I.

In order to identify the potential genetic relationships between Malagasy and international RSSC phylotype I isolates, 145 isolates from a representative worldwide collection (South-West Indian Ocean, Africa, Asia, Americas) kept at the Plant Protection Platform (Saint-Pierre, Réunion, France) were also included in this study (S2 Table).

All RSSC isolates were stored on Cryobank^®^ microbeads (Microbank^®^, PRO-LAB DIAGNOSTICS, Neston, Wirral, U.K.) at -80°C. After growth on nutrient broth and Kelman media [36], 1 μl loop of a bacterial cell suspension was streaked onto agar plates to isolate colonies. Single colonies were suspended in 400 μl of sterile HPLC-grade water and used as templates for PCR amplification.

The phylogenetic assignment of Malagasy phylotype I isolates was based on the multiplex phylotype PCR [29] and the partial nucleotide sequences of the endoglucanase (*egl*) gene [29]. PCR amplification, sequencing and sequevar determination were performed as previously described [37]. The newly generated sequences were deposited in the GenBank database under accession numbers MN310771 to MN310886 and MW014321 to MW014325.

### VNTR identification and selection for MLVA

In a first step, we worked with VNTR loci that had previously been identified throughout the genomes of RSSC isolates belonging to different phylotypes [19, 20, 22, 38]. By using the Muscle algorithm in Geneious v10.0.9 [39], these VNTR, flanked by their primers, were searched and aligned with the reference genome of phylotype I (GMI1000) [40] and nine draft genome sequences of phylotype I isolates: CFBP7058 (sequevar 13), CIR011-208 (sequevar 17), RD13.01 (sequevar 31), TD13.01 (sequevar 31), CIV23 (sequevar 31), TF31.08 (sequevar 31), PSS04 (sequevar 15), TO10 (sequevar 47) and Y45 (sequevar 44) [41, 42]. The purpose of the alignment was to check the presence of the VNTRs in the different genomes of phylotype I and confirm that their characteristics met the following requirements [38]: absence of *indel* in the VNTR, unicity of the VNTR sequence in the genome, non-overlapping VNTR, a very high typeability rate (100% or almost 100%) for phylotype I isolates, 80% internal conservation of the VNTR in the genomes [6], and a repeat unit between 5 and 10 nucleotides [43].

In a second step, we screened the reference GMI1000 genome with the Tandem Repeat Finder v4.09 (https://tandem.bu.edu/trf/trf.html) [44] to detect new VNTR loci. New VNTRs were validated by using the Phobos Tandem Repeat Finder [45]. Then, an alignment with the nine draft genome sequences was conducted to localize the VNTR and confirm whether their characteristics met the requirements described above.

Lastly, to check the distribution and specificity of the selected VNTR markers among RSSC isolates, we screened with BLASTN [46] 114 publicly available RSSC genomes: 48 belonging to phylotype I, 50 to phylotype II, 3 to phylotype III and 13 to phylotype IV (https://www.ncbi.nlm.nih.gov/genome/browse/#!/prokaryotes/490/).

### Primer design and assay optimization

PCR primers were designed from the flanking regions of VNTR loci using Primer3 v4.1.0 [47]. Selection was based on the following criteria: primer size from 18 to 23 bp, annealing temperature between 60°C and 65°C, percentage of guanine and cytosine from 50% to 70%, and total length of PCR product between 100 bp and 500 bp. The probability of dimerization and hairpin were evaluated using the OligoAnalyzer v3.1 software package (https://eu.idtdna.com/calc/analyzer). Oligonucleotides were synthesized by Macrogen, Inc. (Seoul, South Korea).

To perform a preliminary screening, simplex PCR were conducted to assess the amplifiability, reproducibility and polymorphism of the selected VNTR loci. We used eight phylotype I isolates (RUN0054, RUN0157, RUN0215, RUN0334, RUN0969, RUN1744, RUN1985 and RUN3014) representing several sequevars (13, 14, 15, 17, 18, 31 and 47) from a worldwide collection (French Guiana, Réunion, China, Thailand, Cameroon, Taiwan, Côte d’Ivoire). PCR amplifications were performed in 15 μl reaction volumes containing 7.5 μl Terra PCR Direct Buffer 2X (Terra^™^ PCR Direct Polymerase Kit, Clontech Laboratories, Inc.), 0.3 μl Terra PCR Direct Polymerase Mix—1.25 U/μl, 1.5 μl 5X Q-solution (Qiagen^®^, Hilden, Germany), 2.3 μl of a forward and reverse primer mix (2 μM each), 2.4 μl sterile HPLC-grade water, and 1 μl of bacterial suspension as a template. PCRs were carried out in a GeneAmp PCR System 9700 Thermal Cycler (Foster City, CA 94404, USA) under the following conditions: an initial denaturation step at 98°C for 2 min, 30 cycles of denaturation at 98°C for 10s, annealing at 62°C for 15s, extension at 68°C for 1 min, and a final extension step at 68°C for 30 min. Then, 6 μL of PCR product was mixed with 1 ml of loading dye solution and loaded into a 1.5% (w/v) SeaKem^®^ LE Agarose (Lonza, Basel, Switzerland) gel for electrophoresis. Ethidium bromide was used to stain the gels and the G:BOX gel imaging system (Syngene, Cambridge, UK) enabled the visualization of the bands under ultraviolet. The molecular weights were estimated by comparison with a 100 bp DNA ladder (Promega, Madison, Wisconsin, USA). VNTR loci showing poor amplification and/or lacking diversity were removed at this step.

### MLVA genotyping

A Multiplex PCR protocol was applied to analyse from 3 to 4 VNTR loci per reaction using the Multiplex PCR kit (Qiagen, Courtaboeuf, France). The primers (Table 1) were pooled in four different mixes according to their annealing temperature and the size of the PCR product. Each forward primer was marked with one of the fluorophore: 6-FAM, NED, PET and VIC (Applied Biosystems, Life Technologies, Saint Aubin, France). The conditions of amplification for the multiplex PCR were the same as for the simplex PCR, except the number of cycles (25 instead of 30). PCR products were diluted (at least 1:80) to avoid peak saturation.

**Table 1:** Features of the 14 selected VNTR loci composing the RS1-MLVA14 scheme.

Mix	VNTR marker, Locus name^a^	Localization	Code	VNTR sequence	Primers (5’–3’)		Range of repeat numbers^b^^c^	Number of alleles^b^	H_nb_^b^	Allelic richness^b^	Reference
Mix A	GMIch_0581	Intergenic	VNTR01	TGGTT	F:GGGGCGTTGGTGTTTGGCTG	VIC	6–25	14	0.727	3.799	This study
					R:AACACAGGATGCACCACCAG						
	GMIch_2433	Intragenic	VNTR02	CAAGCACCT	F:AGTTCACTGTCGATCCAA	FAM	3–10	8	0.654	4.039	[20]
					R:ACATCCATGTCCGCACGC						
	GMIch_3461	Intragenic	VNTR03	AATGGTTG	F:CGAGGTCGCTCTCCAGAAGGCGA	NED	2–9	6	0.579	2.896	[22]
					R:CGAGAAGGCCAGTCCCGAGCTGA						
	GMIch_1457	intragenic	VNTR04	CGACCGACT	F:CGACCGTCGCCGACGTCC	PET	1–15	13	0.579	3.405	This study
					R:GAGCACGTTGGCGATGAA						
Mix B	GMImp_0487	Intragenic	VNTR06	CACCACGAG	F:CGCAGACGTTCACGAGACTT	FAM	3–5	3	0.359	3.411	This study
					R:CGGACAGCTGTGCCTGATAC						
	GMIch_1844	Intergenic	VNTR07	CGGCATAC	F:CGGCTGAGACCGGATTGC	NED	1–10	10	0.738	4.805	[20]
					R:CTCATGCGAAAGTGTTTGTAAGTGTGT						
	GMImp_1343	Intragenic	VNTR08	GCCCAATCG	F:CACGACATCCACCGCAAG	PET	0^d^–6	7	0.636	3.403	This study
					R:CGGCTCGTTCATCAAGCA						
Mix C	GMImp_0449	Intragenic	VNTR09	CACCACCAG	F:GTAACCCACCCCAACGCTTA	VIC	5–6	2	0.496	2.000	This study
					R:TTGAGCGGATCCTGGTTCTT						
	GMImp_1828	Intergenic	VNTR10	TGCGGA	F:CTCGGGCCGTTCATCGAC	FAM	3–20	16	0.689	5.013	[38]
					R:GATCGGGCAGCCGGATAC						
	GMImp_0740	Intergenic	VNTR12	GACCGCCAC	F:GCTGCATCACTTCGGCATT	PET	2–7	6	0.690	4.227	This study
					R:GAAGCGCAGGGAGATGGTAT						
	GMImp_0618	Intragenic	VNTR13	GCCGCGACA	F:TGCTGTACCGGCTGGCTTA	NED	2–3	2	0.495	2.000	This study
					R:CCAGGCCGAGCAGATCAT						
Mix D	GMImp_0266	Intragenic	VNTR17	CCGGGCAG	F:GGATCGACGTACACGCCTTT	FAM	1–6	6	0.568	2.906	This study
					R:GGACGATGTCGACGTTACGA						
	GMIch_0827	Intergenic	VNTR18	GGCGATGAG	F:TATGTCTCCTGTGCAAGTCGGTG	NED	3–6	4	0.520	2.310	[20]
					R:CCCAAGACCATCTCGGGAAAG						
	GMImp_1618	Intragenic	VNTR19	GGCATCGGC	F:GAATGCCGACGGAAAAACTC	PET	1–2	2	0.027	1.178	This study
					R: TGTCGTGGCACTGGATCTTC						

^**a**^Loci were named according to the genome’s name (GMI = GMI1000), (ch = chromosome, mp = megaplasmid), and physical position (kb).

^**b**^The VNTR number range, the number of alleles per locus, the Nei’s unbiased diversity index (H_nb_), and the allelic richness were calculated from the genotyping data derived from the 293 isolates used in this study.

^**c**^The number of repeats of the VNTR loci was calculated using the following formula:
$$Number\;of\;repeats=\frac{Bin\;size-Forward\;and\;reverse\;primer\;size-Flanking\;region\;size}{VNTR\;size}$$

^**d**^The VNTR was absent in 9 worldwide phylotype I isolates: 4 from Cameroon (RUN0119, RUN0194, RUN0205, RUN0215), 2 from Côte d’Ivoire (RUN1798, RUN1869), 1 from Taiwan (RUN0257), 1 from China (RUN0617), 1 from French Guiana (RUN1972).

In a 2 ml Eppendorf tube, 1080 μl formamide (Hi-Di^™^ formamide, Applied Biosystems) was mixed with 20 μl size marker (GeneScanTM-500 LIZ^®^ Size Standard, Applied Biosystems). Then, in each well of a 96-well plate, 11 μl of the mix was distributed with 1 μl of diluted PCR product. The samples were denatured at 95°C for 5 min, cooled immediately on ice and loaded onto an ABI Prism 3130XL Genetic Analyzer for capillary electrophoresis. The reproducibility of the MLVA genotyping was checked by including 3 phylotype I control isolates in each 96-well plate: RUN0054 (GMI1000, sequevar 18), RUN0157 (PSS04, sequevar 15) and RUN3014 (TFB1.08, sequevar 31).

### Data analysis

The genotyping results were analysed using Geneious v10.0.9. Fragment sizes were estimated using the third order least squares algorithm and attributed to a bin size, which takes into account small size variation due to experimental variation. To confirm the validity and repetition number for each VNTR locus, PCR products of isolates RUN0320, RUN3012, RUN3216 and RUN3277 were sequenced (Genewiz, Leipzig, Germany) and analysed using Geneious v10.0.9. These sequences were also used to check for patterns in the flanking VNTR sequences and internal repeat variations (i.e. copy homology). When a VNTR array was truncated, the VNTR number was rounded to the nearest bin whole number [48–50]. The combination of alleles (i.e. number of repetitions) for the 14 VNTR loci was considered as MLVA type (MT, i.e. haplotype).

In order to assess the resolution of the RS1-MLVA14 scheme, a genotype accumulation curve (GAC) was built, based on the genotyping of 291 phylotype I isolates (146 from Madagascar and 145 from worldwide). The GAC represents the power of discrimination of our set of loci in the population [51, 52]. The discriminatory power of the RS1-MLVA-14 scheme was also evaluated by calculating the Hunter Gaston Discrimination Index (HGDI) [53]. It was compared to the recently developed MLST-7 scheme [37], by using 94 worldwide isolates (S1 and S2 Tables) representing 9 sequevars of phylotype I and selected to maximize the phylogenetic diversity, as well as the diversity of country, host and date of isolation.

Using Arlequin v3.5.2.2 [54], the genetic diversity of Malagasy isolates was evaluated by calculating several indices: Nei’s unbiased estimates of genetic diversity (H_nb_), number of haplotypes, number of alleles, number of private alleles. The allelic richness and private allelic richness were evaluated using HP-RARE [55]. The intra-population diversity indices (H_nb_, number of haplotypes, mean number of alleles, number of private alleles, allelic richness and private allelic richness) provide information on the level of genetic diversity in a population, the population’s epidemiological profile (epidemic or endemic), and the mode of dispersion of the inoculum (stochastic dispersion or dissemination by infected plant material, etc.).

For the robustness of analyses, we considered that a minimum number of samples was required (size n ≥ 14) to form a population. A population is defined a priori by a group of individuals that could exchange genes. Different parameters, such as plant host, altitude and collection area were considered for the analysis of the genetic diversity indices and to define putative populations. The F_ST_ and R_ST_ index calculated with Arlequin (10 000 permutations) provided information on the similarity or differentiation between populations due to the presence or absence of gene flows, mainly due to migration.

We also determined a genetic structure of populations without a priori via the R package Bios2mds [56]. This package is used for conducting metric multidimensional scaling (MDS), a method that represents measurements of similarity (or dissimilarity) among pairs of objects as distances between points of low-dimensional or multidimensional space [57]. Furthermore, the genetic relationships among Malagasy isolates and between Malagasy and worldwide isolates were computed using Phyloviz (http://www.phyloviz.net/) [58]. Different minimum spanning trees (MSTs) were built with the goeBURST full MST algorithm using global optimal eBURST (goeBURST) and Euclidean distances. They revealed the level of differentiation between haplotypes: Single, Double or Triple Locus Variant (SLV, DLV, or TLV), and enabled the identification of clonal complexes (CC). We defined CC as groups of SLV haplotypes in which the founder(s) is (are) defined as the haplotype(s) comprising the largest number of SLV.

## Results

### Development of the RS1-MLVA14 scheme

We developed an optimized MLVA scheme capable of characterizing phylotype I isolates with greater reliability and discriminatory power. Consequently, we were able to overcome certain issues, namely the absence of polymorphism, a low typeability rate and the presence of different VNTRs in some loci [20, 59]. These problems were highlighted by our in-laboratory preliminary tests with Malagasy isolates. Based on previously identified VNTR markers [20, 22, 38] and our present genome screening, we selected a final set of 14 VNTR loci to generate the RS1-MLVA14 scheme (Table 1). VNTR markers were distributed on the chromosome (n = 4) or the megaplasmid (n = 10) (S1 Fig), 5 being intergenic and 9 being intragenic. VNTR sizes varied from 5 to 9 nucleotides and were repeated in the genomes from 1 to 25 times. In our collection of 293 phylotype I isolates, all selected VNTR loci were polymorphic. The number of alleles per locus ranged from 2 to 16 alleles and the allelic richness varied from 1.179 to 5.027. The VNTR marker GMIch_1844 showed the highest genetic diversity (H_nb_ = 0.738), whereas the VNTR marker GMImp_1618 had the lowest (H_nb_ = 0.027). Our screening of 114 publicly available RSSC genomes (S3 Table) revealed that the 14 selected VNTR loci were found in all the 48 phylotype I isolates (except in some incomplete genomes, where 3% of loci could not be found, as already observed [20]). In addition, 10 VNTR loci were found in the 3 phylotype III isolates and 3 to 8 VNTR loci were found in the 50 phylotype II and 13 phylotype IV isolates. Four VNTR loci (GMIch_0581, GMIch_3461, GMIch_1844, GMImp_0266) appeared to be specific to phylotype I.

### Genotypic resolution of the RS1-MLVA14 scheme

The genotypic resolution of our MLVA scheme was assessed by a genotype accumulation curve (S2 Fig), which revealed that our set of loci is sufficient to discriminate between haplotypes in our collection, given that nearly 100% of the genotypes could be detected with 13 markers.

The comparative study of the discriminatory power of RS1-MLVA14 and MLST-7 typing schemes using 94 isolates representing 9 sequevars of phylotype I (S4 Table) showed that RS1-MLVA14 reveals twice as many haplotypes as MLST-7: 27 MLVA Type (MT) instead of 14 Sequence-Type (ST). The discriminatory power is 1.38 times higher with the RS1-MLVA14 than with the MLST-7 (HGDI = 0.793 vs 0.574). RS1-MLVA14 was able to split some sequevars more broadly than MLST-7 (S4 Table). However, it should be noted that both MLST-7 and RS1-MLVA14 schemes were unable to subdivide sequevars I-13, I-14, I-16, I-34, I-46, each of which were represented by only a single ST and a single MT.

### RS1-MLVA14 revealed genetic diversity among Malagasy phylotype I populations and was discriminative at the field scale

The RS1-MLVA14 scheme was applied on 148 Malagasy isolates. The 14 VNTR loci were amplified from all isolates except the GMIch_3461 locus, which was not amplified, despite several assays in two isolates (RUN0306 and RUN0307). Nonetheless, the latter was retained for the analysis of diversity (H_nb_, allelic richness, private allelic richness), genetic structure (F_ST_, R_ST_, MDS) and the genotype accumulation curve (GAC). Overall, 31 haplotypes were identified, including two major haplotypes: MT007 and MT004, which represented 30.14% and 16.44% of the isolates, respectively (S5 Table). The frequency of the other haplotypes varied from 0.68% to 5.48%. Regarding the host of isolation (S3 Fig), the majority of haplotypes (70.97%) were isolated from one host: 32% haplotypes were isolated from *S*. *lycopersicum*, 32% from *C*. *annuum*, 18% from *S*. *aethiopicum*, 14% from *S*. *tuberosum* and 4% from *S*. *melongena*. However, some haplotypes were found on several hosts: the most prevalent haplotype MT007 was found on four hosts (*S*. *aethiopicum*, *S*. *lycopersicum*, *S*. *melongena* and *S*. *tuberosum*); MT015 was found on three hosts (*S*. *aethiopicum*, *S*. *lycopersicum* and *S*. *tuberosum*); and seven haplotypes were found on two hosts, MT004 and MT006 (*S*. *melongena*, *S*. *lycopersicum*), MT009 (*S*. *melongena*, *S*. *scabrum*), MT020 (S. *lycopersicum*, *C*. *annuum*), MT018 and MT022 (*S*. *lycopersicum*, *S*. *tuberosum*) and MT023 (*S*. *nigrum*, *S*. *tuberosum*) (S3 Fig).

**Fig 2:**
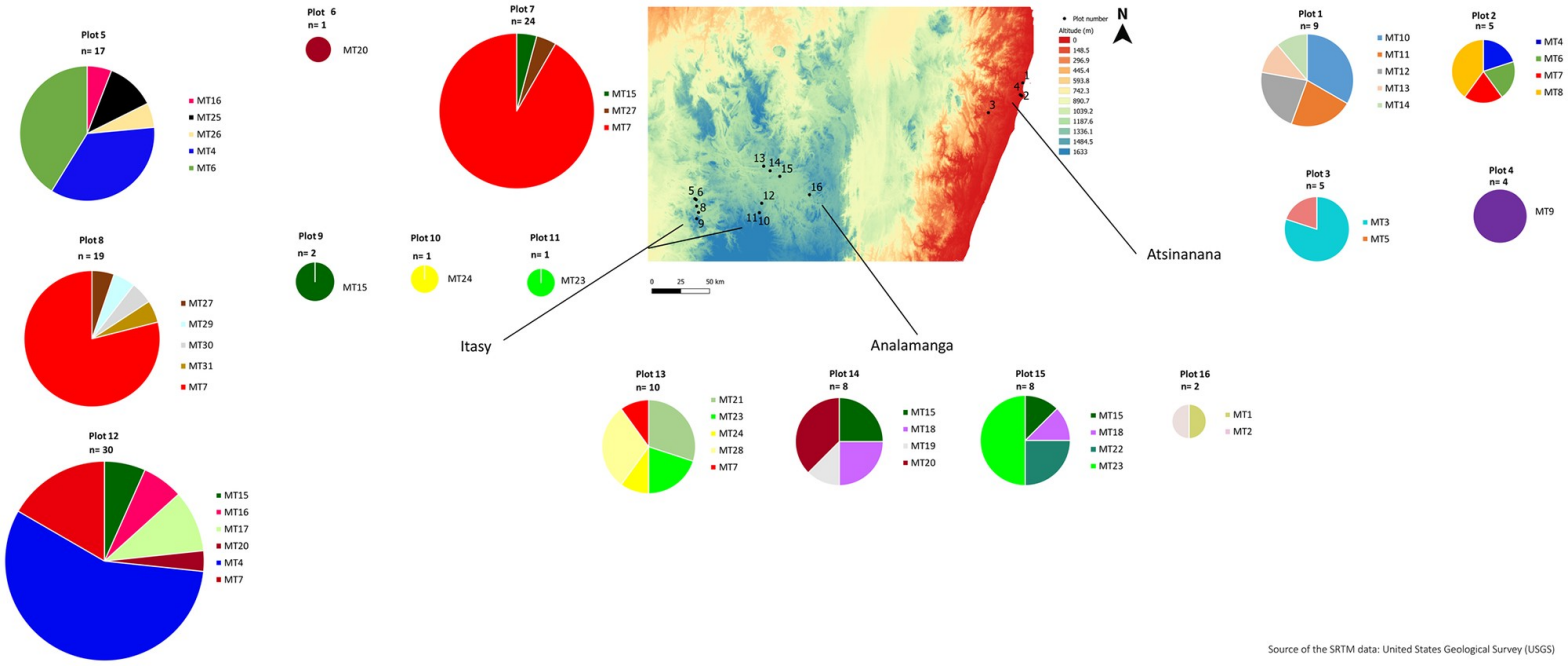
Distribution and frequency of phylotype I haplotypes collected in the 16 fields of the Atsinanana, Analamanga and Itasy regions. The 31 MLVA type (MT) are displayed. Each plot is indicated by a black dot and the number of isolates per plot (n) is mentioned.

The number of haplotypes per field varied from one to six (Fig 2). Interestingly, three haplotypes MT004, MT006 and MT007 were present at both high and low altitudes (Fig 2). The most prevalent haplotype MT007, identified in five fields (Fig 2), was found in the three studied regions (Fig 2). The haplotypes MT004 and MT006, identified in three and two fields, respectively (Fig 2), were found in the regions of Itasy and Atsinanana (Fig 2). Four haplotypes (MT015, MT020, MT023 and MT024) were present in the highland regions of Analamanga and Itasy (Fig 2). The 24 remaining haplotypes were identified in only one region: 9 haplotypes in the Atsinanana region, 8 haplotypes in the Itasy region and 7 haplotypes in the Analamanga region (Fig 2).

The overall genetic diversity of Malagasy phylotype I isolates was H_nb_ = 0.226. However, there were marked differences between the three regions, with a H_nb_ value of 0.109, 0.318, and 0.430 for Itasy, Analamanga and Atsinanana regions, respectively. The average allelic richness over loci and the average private allelic richness over loci also differ between the three regions, 1.54 and 0.05 in Itasy, 2.32 and 0.49 in Analamanga, and 2.98 and 1.28 in Atsinanana (S6 Table).

Genetic differentiation among a priori populations was very important and significant between Itasy and Atsinanana regions (F_ST_ = 0.288***, R_ST_ = 0.381***), important and significant between the Itasy and Analamanga regions (F_ST_ = 0.166***, R_ST_ = 0.252***), and moderate but significant between the Atsinanana and Analamanga regions (F_ST_ = 0.109 ***, R_ST_ = 0.0897*) (S7 Table). An important and significant genetic differentiation (F_ST_ = 0.242***, R_ST_ = 0.336***) was shown between populations from lowlands (0–100 m) and highlands (1200–1500 m) and a moderate but significant differentiation according to the F_ST_ = 0.079*** (R_ST_ value was not significant) between populations from mid-elevation (900–1200 m) and highland areas (1200–1500 m), and between lowland (0–100 m) and mid-elevation (900–1200 m) (F_ST_ = 0.095**; R_ST_ value was not significant) (S7 Table).

### RS1-MLVA14 revealed genetic structure among Malagasy phylotype I populations and showed the singularity of the most prevalent genetic cluster

**Fig 3:**
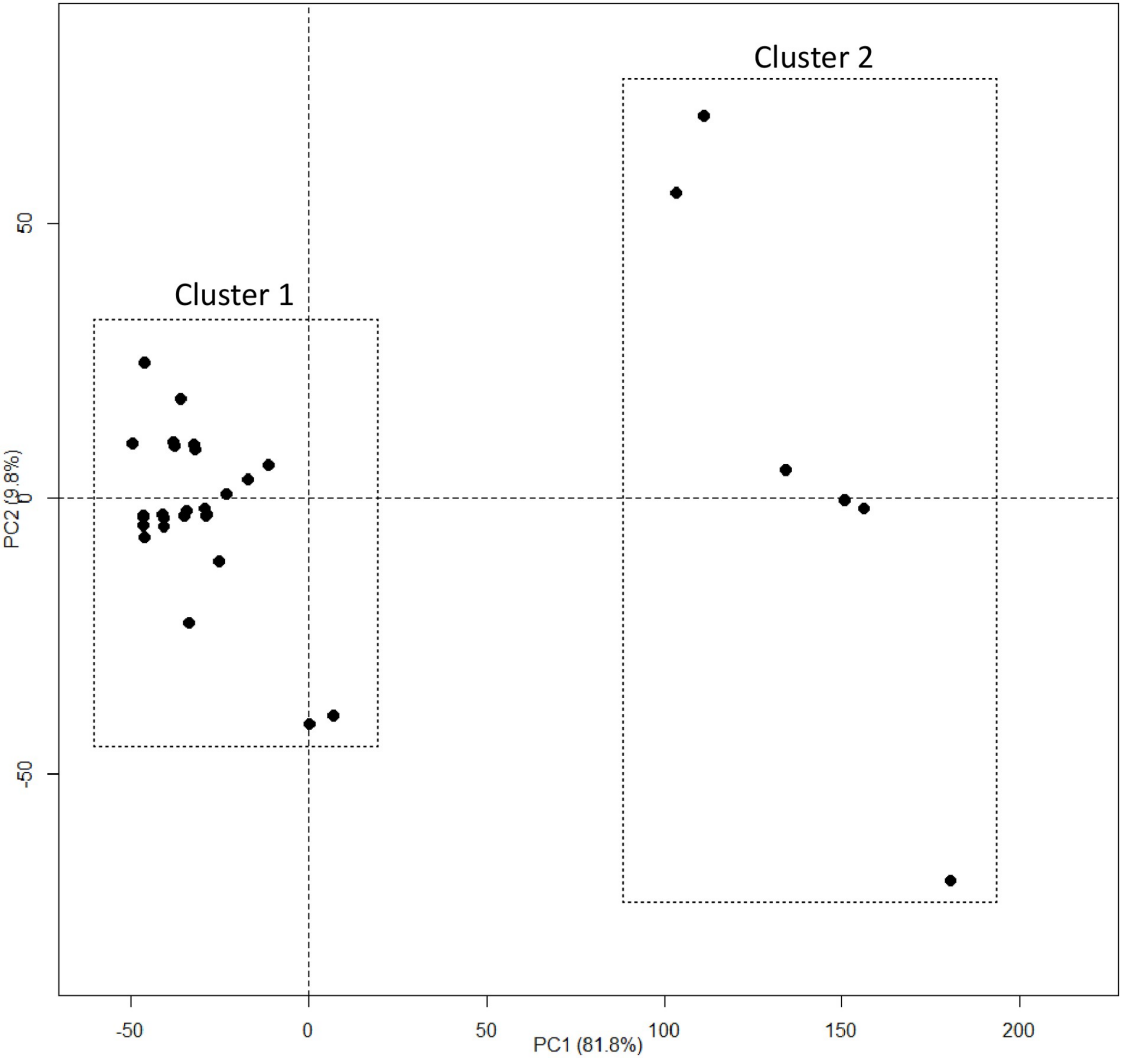
Multidimensional scaling (MDS) of the Malagasy phylotype I (total = 31 haplotypes, 291 isolates). Cluster 1 and cluster 2 represent the two groups of haplotypes.

The MDS representation showed that the Malagasy haplotypes could be grouped into two clusters with a probability of 67.5% (Fig 3). Cluster 1 and cluster 2 included 25 and 6 haplotypes, respectively. Haplotypes were assigned to these clusters with high probability (90.9%). Axis 1 and Axis 2 explained 81.7% and 9.2% of the variance, respectively (Fig 3).

**Fig 4:**
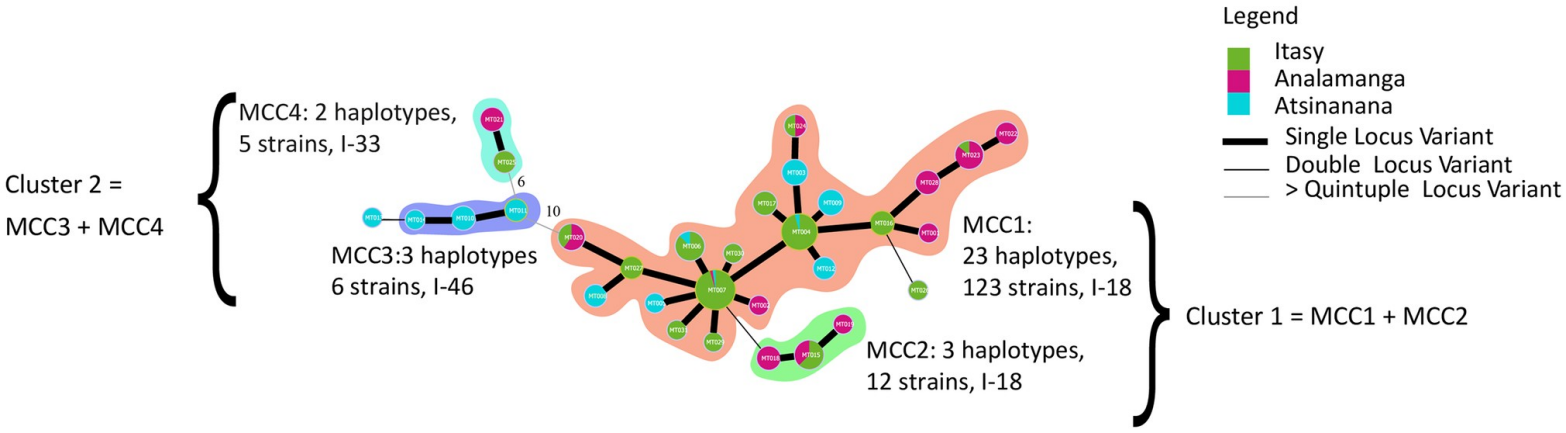
Minimum spanning tree (MST) of the Malagasy phylotype I as a function of the region where samples were collected. The haplotypes were identified by using goeBURST full MST in PHYLOVIZ. Each MLVA type (MT) is displayed as a circle, the size of which is proportional to the number of isolates represented. The different colours indicate the region where data was collected. The branch thickness indicates the number of locus differences between the neighbouring haplotypes. MCC1, MCC2, MCC3, MCC4 represent the Malagasy Clonal Complexes 1, 2, 3 and 4. A clonal complex is composed of haplotypes that differ only by one VNTR locus. I-18, I-33 and I-46 represents the sequevar of the isolates.

A network representation (MST) revealed the genetic relationships between the 31 Malagasy haplotypes (Fig 4). A major Malagasy clonal complex (MCC1), composed of isolates that differed by only one VNTR, was identified. MCC1 gathered 23 haplotypes (cluster 1, 123 isolates), from which two founder haplotypes (the most prevalent haplotypes, MT007 and MT004) were predicted. Three minor Malagasy clonal complexes were identified: MCC2 (cluster 1, 3 haplotypes, 12 isolates), MCC3 (cluster 2, 3 haplotypes, 6 isolates), and MCC4 (cluster 2, 2 haplotypes, 5 isolates).

Interestingly, the MDS and MST representations were congruent with the phylogenetic assignment (i.e. sequevar) of the isolates based on *egl* partial sequences. Indeed, cluster 1 (MCC1, MCC2) grouped the isolates belonging to the sequevar 18, while cluster 2 gathered isolates belonging to sequevar 33 (MCC4) and sequevar 46 (MCC3). Moreover, MCC1 was the only CC which included isolates isolated from both highlands and lowlands. The remaining CCs comprised isolates isolated from the highlands only (MCC2, MCC4) or from the lowlands only (MCC3) (Fig 4). All CCs included isolates collected from several hosts except MCC3, for which isolates were only collected from *C*. *annuum* (S3 Fig).

**Fig 5:**
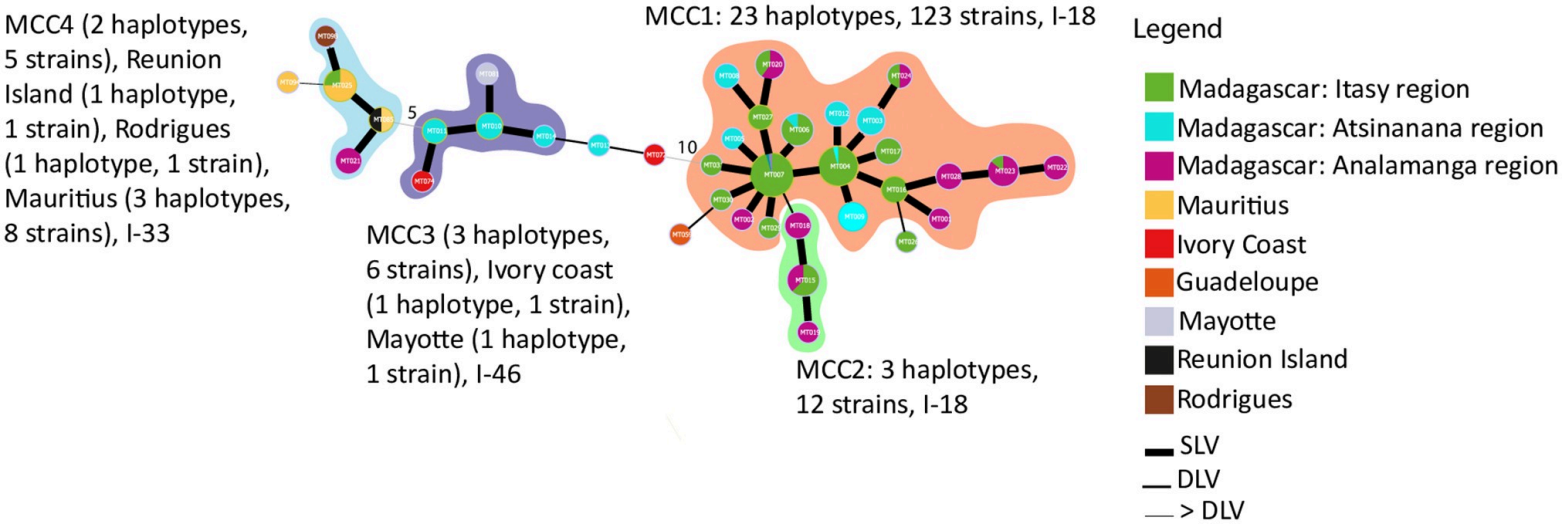
Minimum spanning tree (MST) of the Malagasy and worldwide phylotype I. The haplotypes were identified by using goeBURST full MST in PHYLOVIZ. Each MLVA type (MT) is displayed as a circle, the size of which is proportional to the number of isolates represented. The different colours indicate the country. The branch thickness indicates the number of locus differences between the neighbouring haplotypes. MCC1, MCC2, MCC3, MCC4 represent the Malagasy Clonal Complex 1, 2, 3 and 4. A clonal complex is composed of haplotypes that differ only by one VNTR locus. The sequevar of the isolates are represented as I-18, I-33 and I-46.

A global MST was built to display the genetic relationships between phylotype I isolates from Madagascar (146 isolates, 31 haplotypes) and from worldwide isolates (145 isolates, 76 haplotypes) (Fig 5). Only the seven worldwide haplotypes, which had genetic links with the 31 Malagasy haplotypes, are shown in Fig 5.

The global MST revealed 4 clonal complexes. Strikingly, Malagasy haplotypes in cluster 1 (MCC1, MCC2), corresponding to sequevar 18, appeared genetically distant from worldwide phylotype I isolates. Indeed, the major Malagasy MCC1 differed to international haplotypes by at least ten loci (except one strain from Guadeloupe, MT059, which differed by 2 loci). The Malagasy minor MCC2 appeared closely related to MCC1 and unrelated to other worldwide haplotypes. In contrast, Malagasy haplotypes in cluster 2 (MCC3 and MCC4, which correspond to sequevars 46 and 33, respectively) appeared related to other worldwide haplotypes. Indeed, the three Malagasy haplotypes (MT010, MT011, and MT014) belonging to MCC3 were very closely related to haplotypes from Mayotte and Côte d’Ivoire (single-locus variant). Thus, they were grouped in the same CC. Interestingly, the haplotypes from Madagascar (MT010, MT011 and MT014) and Mayotte (MT081) were isolated from the same host species, *C*. *annuum*. The two Malagasy haplotypes, MT021 and MT025, belonging to MCC4 were included in a CC, which gathered isolates from Rodrigues, Mauritius and Réunion. Interestingly, within this CC, the haplotype MT025 isolated from *S*. *lycopersicum* was found both in Mauritius and in Madagascar. *S*. *tuberosum* was also the host plant shared by the haplotypes collected in Madagascar (MT021) and Mauritius (MT025, MT094).

## Discussion

A growing interest in the use of MLVA on plant pathogenic bacteria for studying population biology has been observed in recent years [4, 11, 13–17]. As far as the RSSC is concerned, MLVA schemes have been applied to the global surveillance of phylotype III [38], to trace potential sources of contamination by phylotype IIB-1 in England and Ethiopia [21, 60]. Very recently, they have been used to study the genetic diversity and structure of phylotypes I and IIB-1 populations in Uganda [59]. The study of RSSC populations (phylotypes I, II and III) collected in the central highlands of Madagascar in 2013 was the first project of such magnitude (1224 isolates collected) [19]. Remarkably, this work revealed contrasting epidemiological patterns between phylotype IIB-1 and phylotype III populations. The population biology of Malagasy phylotype I had not yet been studied due to the absence of reliable MLVA markers to amplify phylotype I isolates [19]. The objective of the present study was to: develop and apply an optimized MLVA scheme to study the population genetics of Malagasy phylotype I; analyse the genetic relationships with worldwide phylotype I; and compare the epidemiological situation of phylotype I with Malagasy phylotypes IIB-1 and III.

### RS1-MLVA14 scheme, a genotyping tool adapted to study the population genetics of phylotype I, both at field and global scales

By using previously identified VNTR markers [20, 22, 38], as well as by screening genomes for new VNTR markers, we developed an optimized MLVA scheme adapted to RSSC phylotype I isolates. The 14 selected VNTR markers were all present in phylotype I, to a lesser extent in phylotype III and rarely found in phylotypes II and IV. This is consistent with the species delineation of phylotypes I and III (belonging to *R*. *pseudosolanacearum*) compared to the phylotype II (*R*. *solanacearum*) and phylotype IV (*R*. *syzygii*) [27, 28]. The RS1-MLVA14 scheme showed a very high typeability because only one VNTR could not be amplified from 2 out the 293 phylotype I isolates tested. We assessed whether the RS1-MLVA14 scheme improved genotypic resolution compared to MLST-7 [37]. MLST is the gold standard for epidemiological surveillance and outbreak investigations of bacterial diseases. The value of the discriminatory power of the RS1-MLVA14 scheme (HGDI = 0.793) was higher than for the MLST-7 scheme (HGDI = 0.574). This revealed greater genetic and haplotypic diversity and highlighted the interest of this new MLVA scheme. VNTR loci with low diversity values are useful to establish phylogenetic relationships. Those with a high diversity have a strong discriminatory power [61]. VNTR are supposed to have high mutation rates, which increases the likelihood of homoplasy [62]. Another point is that molecular samples with lower values of HGDI may indicate high levels of homoplasy. However, low values may also be due to low mutation rates [62]. The RS1-MLVA14 scheme combines VNTR loci with variable diversity values and retains the phylogenetic signal, since our MDS and MST analyses showed that the isolates were grouped according to their phylogenetic assignment (sequevar). These results suggest that the probability of homoplasy is low. They support previous studies, such as the work on phylotype III of the RSSC [38] or other plant pathogens [3, 17]. Lastly, we showed that the RS1-MLVA14 scheme can be used to explore the genetic diversity at the field scale (several haplotypes were disclosed in single fields), as well as at a global scale. Thus, RS1-MLVA14 is well suited to deciphering the epidemiology of phylotype I populations.

### RS1-MLVA14 unveiled contrasting genetic diversity and epidemiology among Malagasy phylotype I populations

The analysis of phylotype I populations revealed contrasting genetic diversity depending on the three Malagasy regions. Genetic diversity is greater in the lowlands than in highland areas. In the lowlands, the Atsinanana region imports huge amounts of vegetables from other Malagasy producing areas, such as Ambatondrazaka or wholesale markets in Antananarivo, the Malagasy capital [63]. In addition, this region has the Toamasina seaport, where there is a great deal of trade in plant material with foreign countries, which could be responsible for the introduction of contaminated agricultural products in this lowland region. It might also explain why the level of genetic diversity of phylotype I isolates is greater.

Our findings showed a lower genetic diversity in phylotype I populations in the central Malagasy highlands. Some haplotypes were found in different fields in the same region (for example MT007 was detected in plots 7, 9 and 12 in the Itasy region), which suggests possible field contamination from exchanges of infected plant material. Indeed, producers do not always have access to healthy seeds [64]. Farmers often produce their own seeds or exchange seeds with other farmers, with no sanitary guarantee. Trade in agricultural products might encourage the propagation of haplotypes between regions. Most vegetables from the Itasy region are sold in the capital, Antananarivo (Analamanga region), at Madagascar’s wholesale markets, where large volumes of agricultural products are traded [64].

Another interesting point shown by our study is that Malagasy phylotype I populations (sequevars 18, 33 and 46) are genetically differentiated depending on the elevation. Remarkably, a similar situation was observed in China, where the genetic structure of phylotype I isolates (for sequevars 13, 14, 15, 17, 34, 44, 54 and 55) was associated with elevation [65]. Sequevar 18 seems to be adapted to both warm and cool temperatures in Madagascar, since it was isolated in the lowlands at sea level in the Atsinanana region (temperature range is from 17°C to 30.1°C), as well as in the highlands at an altitude of up to 1500m in the Analamanga region (from 8.9°C to 26.6°C) and the Itasy region (from 9.1°C to 28°C) [66]. Interestingly, sequevar 31 isolated in the lowlands and up to altitudes above 1000m in Réunion [37], as well as in Côte d’Ivoire [67], exhibited a similar environmental distribution. So far, sequevar 18 has always been reported from lowlands in the Americas [68–70], Africa [67], Asia [71–73], Oceania [74] and on islands in the South-West Indian Ocean [37, 75]. In contrast, even though a very limited number of isolates was used in our study, sequevar 33 was only isolated at moderate and high elevations (above 900m) and sequevar 46 was only isolated in lowlands (below 300m). Thus, in addition to the studies in China [65], Réunion [37] and Côte d’Ivoire [67], our study in Madagascar shed new light on the ecology of phylotype I isolates, which are often reported as only being composed of tropical and subtropical isolates adapted to lowland areas. Altogether, the ecology of phylotype I isolates differs from that of phylotype IIB-1 and phylotype III isolates, which are considered as cold-tolerant and adapted to highland areas [19, 23, 24, 76, 77]. Further research and massive sampling of phylotype I isolates would certainly provide a better understanding of the differences in the prevalence of sequevars according to elevation and the factors determining their differential environmental adaptation.

Our study of population structure showed that Malagasy phylotype I isolates were distributed in two genetic clusters, which displayed different epidemiological features. A striking result is the singularity of the most prevalent Malagasy isolates belonging to sequevar 18 (corresponding to cluster 1). Indeed, despite the fact that sequevar 18 is distributed worldwide [37, 67–73, 75], our study revealed no genetic links between Malagasy isolates and worldwide isolates (South-West Indian Ocean, Africa, Americas, Asia isolates). The closest haplotype (one strain) was a double-locus variant from Guadeloupe. Cluster 1 could be derived from the evolution of an ancient introduction of isolates that evolved locally. This would explain the differentiation from worldwide isolates, with short-distance dissemination restricted to Madagascar. This work must be continued by integrating more Malagasy and global phylotype I isolates to further our understanding of the singularity of isolates belonging to cluster 1. In contrast, cluster 2, which included the least prevalent isolates in Madagascar (sequevars 33 and 46), showed genetic links with isolates from South-West Indian Ocean islands (Mayotte, Mauritius, Rodrigues and Réunion). So far, apart from the recent introduction of phylotype I via rose cuttings in the Netherlands [78], no long-distance dissemination of phylotype I isolates has been reported. Only the spread of phylotype IIB isolates via bananas, potato tubers and geranium cuttings [79–81] has been clearly documented. Interestingly, based on the *egl* sequence analysis, the phylotype I isolates introduced in the Netherlands were assigned to sequevar 33 [82], the sequevar that is likely to have been disseminated between Madagascar and other South-West Indian Ocean islands. Moreover, the *egl* sequences from the Dutch isolates appeared 100% identical to *egl* sequences from isolates from Madagascar, Mauritius [37], Rodrigues [37, 83] and India [84]. This highlights the phylogenetic links between isolates from distant geographical areas. As far as the sequevar 46 is concerned, it has now been reported in Madagascar and Mayotte (South-West Indian Ocean) [75], as well as in more geographically distant areas, such as Côte d’Ivoire [67] and Myanmar [85]. All these data strongly support the theory of the global dissemination of phylotype I, which is probably linked to human activities and the transport of contaminated material [86].

### Comparative epidemiology of Malagasy phylotype I, IIB-1 and III populations

Our study showed that the level of genetic diversity of Malagasy phylotype I populations was globally low (H_nb_ = 0.226). This result was surprising since, according to McDonald and Linde [1], phylotype I is considered to have a high evolutionary potential due to its natural ability to transform and recombine [37, 86, 87]. The level of genetic diversity of Malagasy phylotype I populations was quite similar to that of Malagasy phylotype IIB-1 populations (H_nb_ = 0.19) and clearly lower than that of Malagasy phylotype III populations (H_nb_ = 0.40) [19]. As determined for Malagasy phylotype IIB-1 [19], the Malagasy phylotype I haplotypes appeared to be closely related genetically and gathered into clonal complexes. Some haplotypes were shared between fields in the same agroecological region and even between agroecological areas (at low and high elevations). In contrast to the endemic nature of phylotype III [19], these results suggest that the phylotype I isolates have a similar epidemic population pattern to phylotype IIB-1 isolates.

In Madagascar’s central highlands above 1000 m elevation, phylotype I populations co-occurred with populations of phylotype IIB-1 and III [19]. In addition, as for phylotypes IIB-1 and III [19], phylotype I populations were isolated from many solanaceous crops (*S*. *lycopersicum*, *S*. *tuberosum*, *S*. *melongena*, *S*. *gilo* and *C*. *annuum*), and weeds (*S*. *nigrum*, *S*. *scabrum*). Weeds play an important role. They often remain in the soil after harvesting and harbour pathogenic populations, which survive in the environment as a result [24, 88, 89]. Our data supports this because although our weed sampling was very limited, RS1-MLVA14 did not reveal specific haplotypes on weeds. However, in the central Malagasy highlands, phylotype I populations were the least prevalent (10% instead of 18% for phylotype III and 72% for phylotype IIB-1) [19]. This observation could be explained by the fact that phylotype IIB-1 populations are reported to be better adapted to low temperatures compared to phylotype III populations and even more so compared to phylotype I populations [76, 77, 90]. Interestingly, a comparative pathogenicity test of Malagasy phylotypes I, IIB-1 and III isolates on eight potato varieties showed that all isolates were strongly pathogenic at tropical lowland temperatures, but that IIB-1 isolates were the most aggressive [18]. Similar comparative pathogenicity tests should now be performed on different hosts at cold temperatures to determine whether temperature influences the capacity of the three phylotype isolates to co-occur in the same cropping areas and cause disease.

In conclusion, during this study, we developed an optimized MLVA scheme dedicated to phylotype I populations. We showed that the RS1-MLVA14 scheme is highly resolutive at global, regional and field scales, which makes it suitable for epidemiological studies. This work on Malagasy phylotype I represents a first step. Our research was limited to three vegetable growing regions in Madagascar. A broader study has been launched with a wider spatial scale, including all the vegetable cropping areas in Madagascar. The goal is to further our understanding of the migration routes and population biology of phylotype I in Madagascar and between Madagascar and other countries.

## Supporting information

S1 Table Characteristics of the 148 Malagasy isolates used in the study. The isolates used for the comparative analysis of MLST-7 and RS1-MLVA14 are in bold.
(XLSX)

S2 Table Characteristics of the 145 worldwide isolates used in the study. The isolates used for the comparative analysis of MLST-7 and RS1-MLVA14 are in bold.
(XLSX)

S3 Table Screening of the 14 selected VNTR loci in the 114 publicly available RSSC genomes. (XLSX)

S4 Table Correspondence between sequevar, ST and haplotype for the comparative analysis of MLST-7 and RS1-MLVA14. (XLSX)

S5 Table Frequency of the Malagasy MLVA type. (XLSX)

S6 Table Diversity intra-population indexes. (XLSX)

S7 Table F_ST_ and R_ST_ between populations from the three regions where data was collected and between populations from lowlands, mid-elevation and highlands. (XLSX)

S1 Fig Localization of the 14 VNTRs on the chromosome and megaplasmid of the reference phylotype I strain GMI1000. (TIF)

S2 Fig Genotype accumulation curve for 291 strains of RSSC phylotype I strains genotyped over 14 loci. The horizontal axis represents the number of loci randomly sampled without replacement up to n-1 loci, the vertical axis shows the number of unique MLVA types observed (n = 107) in the data set. The level of 100% of unique MLVA types detected is indicated with a dotted red line.
(TIF)

S3 Fig Minimum spanning tree (MST) of the Malagasy phylotype I according to the host of collect. The haplotypes were identified by using goeBURST full MST in PHYLOVIZ. Each MLVA type (MT) is displayed as a circle, the size of which is proportional to the number of isolates represented. The different colours indicate the sample host. Branch thickness indicates the number of locus differences between the neighbouring haplotypes. MCC1, MCC2, MCC3, MCC4 represent the Malagasy Clonal Complexes 1, 2, 3 and 4. A clonal complex is composed of haplotypes that differ only by one VNTR locus. I-18, I-33 and I-46 represent the phylotype-sequevar of the isolates.
(TIF)

## References

[1] McDonald BA, Linde C. Pathogen population genetics, evolutionary potential, and durable resistance. Annu Rev Phytopathol. 2002;40: 349–379. 10.1146/annurev.phyto.40.120501.101443 12147764

[2] Milgroom MG. Population biology of plant pathogens: genetics, ecology, and evolution. The American Phytopathological Society; 2017.

[3] López-Soriano P, Boyer K, Cesbron S, Morente MC, Peñalver J, Palacio-Bielsa A, et al Multilocus Variable Number of Tandem Repeat Analysis reveals multiple introductions in Spain of *Xanthomonas arboricola pv*. *pruni*, the causal agent of bacterial spot disease of stone fruits and almond. Vinatzer BA, editor. PLOS ONE. 2016;11: e0163729 10.1371/journal.pone.0163729 27669415 PMC5036818

[4] Bühlmann A, Dreo T, Rezzonico F, Pothier JF, Smits THM, Ravnikar M, et al Phylogeography and population structure of the biologically invasive phytopathogen *Erwinia amylovora* inferred using minisatellites. Environ Microbiol. 2014;16: 2112–2125. 10.1111/1462-2920.12289 24112873

[5] Mascheretti S, Croucher PJP, Vettraino A, Prospero S, Garbelotto M. Reconstruction of the sudden oak death epidemic in California through microsatellite analysis of the pathogen *Phytophthora ramorum*: genetic structure of *P*.*ramorum* in California. Mol Ecol. 2008;17: 2755–2768. 10.1111/j.1365-294X.2008.03773.x 18444982

[6] Vergnaud G, Pourcel C. Multiple Locus Variable Number of Tandem Repeats Analysis In: Caugant DA, editor. Molecular epidemiology of microorganisms. Totowa, NJ: Humana Press; 2009 pp. 141–158. 10.1007/978-1-60327-999-4_12 19521873

[7] Pourcel C, Vergnaud G. Strain typing using Multiple "Variable Number of Tandem Repeat” Analysis and genetic element CRISPR. 2011.

[8] Van Belkum A. Tracing isolates of bacterial species by Multilocus Variable number of tandem repeat Analysis (MLVA). FEMS Immunol Med Microbiol. 2007;49: 22–27. 10.1111/j.1574-695X.2006.00173.x 17266711

[9] Zaluga J, Stragier P, Van Vaerenbergh J, Maes M, De Vos P. Multilocus Variable Number Tandem Repeats Analysis (MLVA) distinguishes a clonal complex of *Clavibacter michiganensis subsp*. *michiganensis* strains isolated from recent outbreaks of bacterial wilt and canker in belgium. BMC Microbiol. 2013;13: 126 10.1186/1471-2180-13-126 23738754 PMC3691591

[10] Gironde S, Manceau C. Housekeeping gene sequencing and Multilocus Variable Number Tandem Repeat Analysis to identify subpopulations within *Pseudomonas syringae pv*. *maculicola* and *Pseudomonas syringae pv*. *tomato* that correlate with host specificity. Appl Environ Microbiol. 2012;78: 3266–3279. 10.1128/AEM.06655-11 22389364 PMC3346470

[11] Francisco CS, Ceresini PC, Almeida RPP, Coletta-Filho HD. Spatial genetic structure of coffee associated *Xylella fastidiosa* populations indicates that cross infection does not occur with sympatric citrus orchards. Phytopathology. 2017;107: 395–402. 10.1094/PHYTO-08-16-0300-R 27992307

[12] Bui Thi Ngoc L, Verniere C, Jarne P, Brisse S, Guerin F, Boutry S, et al From local surveys to global surveillance: three high-throughput genotyping methods for epidemiological monitoring of *Xanthomonas citri pv*. *citri* pathotypes. Appl Environ Microbiol. 2009;75: 1173–1184. 10.1128/AEM.02245-08 19088309 PMC2643580

[13] Pruvost O, Magne M, Boyer K, Leduc A, Tourterel C, Drevet C, et al A MLVA genotyping scheme for global surveillance of the Citrus Pathogen *Xanthomonas citri pv*. *citri* suggests a worldwide geographical expansion of a single genetic lineage. Vinatzer BA, editor. PLoS ONE. 2014;9: e98129 10.1371/journal.pone.0098129 24897119 PMC4045669

[14] Poulin L, Grygiel P, Magne M, Gagnevin L, Rodriguez-R LM, Forero Serna N, et al New Multilocus Variable Number Tandem Repeat Analysis tool for surveillance and local epidemiology of bacterial leaf blight and bacterial leaf streak of rice caused by *Xanthomonas oryzae*. Goodrich-Blair H, editor. Appl Environ Microbiol. 2015;81: 688–698. 10.1128/AEM.02768-14 25398857 PMC4277570

[15] Rache L, Blondin L, Flores C, Trujillo C, Szurek B, Restrepo S, et al An Optimized Microsatellite Scheme for Assessing Populations *of Xanthomonas phaseoli* pv. *manihotis*. Phytopathology. 2019;109: 859–869. 10.1094/PHYTO-06-18-0210-R 30908143

[16] Nakato GV, Fuentes Rojas JL, Verniere C, Blondin L, Coutinho T, Mahuku G, et al A new Multi Locus Variable Number of Tandem Repeat Analysis scheme for epidemiological surveillance of *Xanthomonas vasicola pv*. *musacearum*, the plant pathogen causing bacterial wilt on banana and enset. Chiang T-Y, editor. PLOS ONE. 2019;14: e0215090 10.1371/journal.pone.0215090 30973888 PMC6459536

[17] Ferreira MASV, Bonneau S, Briand M, Cesbron S, Portier P, Darrasse A, et al *Xanthomonas citri pv*. *viticola* affecting grapevine in Brazil: emergence of a successful monomorphic pathogen. Front Plant Sci. 2019;10: 489 10.3389/fpls.2019.00489 31057588 PMC6482255

[18] Ravelomanantsoa S. Biologie des populations du complexe d’espèces Ralstonia solanacearum appliquée à l’épidémiologie du flétrissement bactérien de la pomme de terre à Madagascar. Université de La Réunion; 2016.

[19] Ravelomanantsoa S, Vernière C, Rieux A, Costet L, Chiroleu F, Arribat S, et al Molecular epidemiology of bacterial wilt in the Madagascar highlands caused by Andean (Phylotype IIB-1) and African (Phylotype III) brown rot strains of the *Ralstonia solanacearum* species complex. Front Plant Sci. 2018;8 10.3389/fpls.2017.02258 29379515 PMC5775269

[20] N’Guessan CA, Brisse S, Le Roux-Nio A-C, Poussier S, Koné D, Wicker E. Development of variable number of tandem repeats typing schemes for *Ralstonia solanacearum*, the agent of bacterial wilt, banana Moko disease and potato brown rot. J Microbiol Methods. 2013;92: 366–374. 10.1016/j.mimet.2013.01.012 23376194

[21] Parkinson N, Bryant R, Bew J, Conyers C, Stones R, Alcock M, et al Application of Variable-Number Tandem-Repeat Typing to discriminate *Ralstonia solanacearum* strains associated with English watercourses and disease outbreaks. Appl Environ Microbiol. 2013;79: 6016–6022. 10.1128/AEM.01219-13 23892739 PMC3811358

[22] Guinard J, Latreille A, Guérin F, Poussier S, Wicker E. New Multilocus Variable Number Tandem Repeat Analysis (MLVA) scheme for fine scale monitoring and microevolution related study of *Ralstonia pseudosolanacearum* phylotype I populations. Drake HL, editor. Appl Environ Microbiol. 2017;83 10.1128/AEM.03095-16 28003195 PMC5311393

[23] Elphinstone J. The current bacterial wilt situation: a global overview. Bacterial wilt disease and the Ralstonia solanacearum species complex. 2005; 9–28.

[24] Hayward AC. The hosts of Pseudomonas solanacearum. Bacterial wilt: the disease and its causative agent, Pseudomonas solanacearum. 1994; 9–24.

[25] Mansfield J, Genin S, Magori S, Citovsky V, Sriariyanum M, Ronald P, et al Top 10 plant pathogenic bacteria in molecular plant pathology: Top 10 plant pathogenic bacteria. Mol Plant Pathol. 2012;13: 614–629. 10.1111/j.1364-3703.2012.00804.x 22672649 PMC6638704

[26] Prior P, Fegan M. Recent developments in the phylogeny and classification of *Ralstonia solanacearum*. Acta Hortic. 2005; 127–136. 10.17660/ActaHortic.2005.695.14

[27] Prior P, Ailloud F, Dalsing BL, Remenant B, Sanchez B, Allen C. Genomic and proteomic evidence supporting the division of the plant pathogen *Ralstonia solanacearum* into three species. BMC Genomics. 2016;17: 90 10.1186/s12864-016-2413-z 26830494 PMC4736150

[28] Safni I, Cleenwerck I, De Vos P, Fegan M, Sly L, Kappler U. Polyphasic taxonomic revision of the *Ralstonia solanacearum* species complex: proposal to emend the descriptions of *Ralstonia solanacearum* and *Ralstonia syzygii* and reclassify current *R*. *syzygii* strains as *Ralstonia syzygii subsp*. *syzygii subsp*. *nov*., *R*. *solanacearum* phylotype IV strains as *Ralstonia syzygii subsp*. *indonesiensis subsp*. *nov*., banana blood disease bacterium strains as *Ralstonia syzygii subsp*. *celebesensis subsp*. *nov*. and *R*. *solanacearum* phylotype I and III strains as *Ralstonia pseudosolanacearum sp*. *nov*. Int J Syst Evol Microbiol. 2014;64: 3087–3103. 10.1099/ijs.0.066712-0 24944341

[29] Fegan M, Prior P. How complex is the “Ralstonia solanacearum Species Complex.” APS Press. Bacterial wilt disease and the Ralstonia solanacearum species complex. APS Press. Minnesota: Allen, C., Prior, P., Hayward, A.C.; 2005. pp. 449–461.

[30] Bouriquet G. Les maladies du tabac à Madagascar. Annu Cryptogam Exot. 1934;VII: 97–112.

[31] Bouriquet G. Madagascar: list of the parasites and diseases of cultivated plants. Int Bull Plant Prot. 1934;VIII: 99–100.

[32] Bouriquet G. Les maladies des plantes cultivées à Madagascar. Encycl Mycol. 1946;XII: 545.

[33] Dadant R, Rasolofo R, Baudin P. Liste des maladies des plantes cultivées à Madagascar. 1960;17: 94.

[34] Lallmahomed GM, Rakotobe Rabehevitra E, Rakotondramanana. Biovars and races of *Pseudomonas solanacearum* in Madagascar a preliminary study. FAO Plant Prot Bull. 1988;36: 54–59.

[35] Rabakoarihanta A, Rakotondramanana. “Potato production status in Madagascar,” in Potato development and transfer of technology in tropical africa. International Potato Center. Nairobi—Kenya; 1984.

[36] Kelman A. The relationship of pathogenicity of *Pseudomonas solanacearum* to colony appearance in a tetrazolium medium. Phytopathology. 1954;54: 693–695. 10.1094/Phyto-44-693 40934411

[37] Yahiaoui N, Chéron J-J, Ravelomanantsoa S, Hamza AA, Petrousse B, Jeetah R, et al Genetic diversity of the *Ralstonia solanacearum* species complex in the South-West Indian Ocean islands. Front Plant Sci. 2017;8: 2139 10.3389/fpls.2017.02139 29312394 PMC5742265

[38] Ravelomanantsoa S, Robène I, Chiroleu F, Guérin F, Poussier S, Pruvost O, et al A novel multilocus variable number tandem repeat analysis typing scheme for African phylotype III strains of the *Ralstonia solanacearum* species complex. PeerJ. 2016;4: e1949 10.7717/peerj.1949 27168969 PMC4860299

[39] Kearse M, Moir R, Wilson A, Stones-Havas S, Cheung M, Sturrock S, et al Geneious Basic: An integrated and extendable desktop software platform for the organization and analysis of sequence data. Bioinformatics. 2012;28: 1647–1649. 10.1093/bioinformatics/bts199 22543367 PMC3371832

[40] Salanoubat M, Genin S, Artiguenave F, Gouzy J, Mangenot S, Arlat M, et al Genome sequence of the plant pathogen *Ralstonia solanacearum*. Nature. 2002;415: 497–502. 10.1038/415497a 11823852

[41] Li Z, Wu S, Bai X, Liu Y, Lu J, Liu Y, et al Genome sequence of the tobacco bacterial wilt pathogen *Ralstonia solanacearum*. J Bacteriol. 2011;193: 6088–6089. 10.1128/JB.06009-11 21994922 PMC3194909

[42] Guinard J, Vinatzer BA, Poussier S, Lefeuvre P, Wicker E. Draft genome sequences of nine strains of *Ralstonia solanacearum* differing in virulence to eggplant (*Solanum melongena*). Genome Announc. 2016;4: e01415–01415. 10.1128/genomeA.01415-15 26823572 PMC4732325

[43] Mrazek J, Guo X, Shah A. Simple sequence repeats in prokaryotic genomes. Proc Natl Acad Sci. 2007;104: 8472–8477. 10.1073/pnas.0702412104 17485665 PMC1895974

[44] Benson G. Tandem repeats finder: a program to analyze DNA sequences. Nucleic Acids Res. 1999;27: 573–580. 10.1093/nar/27.2.573 9862982 PMC148217

[45] Mayer C. Phobos: a tandem repeat search tool. 2007.

[46] Altschul SF, Gish W, Miller W, Myers EW, Lipman DJ. Basic Local Alignment Search Tool. J Mol Biol. 1990;215: 403–410. 10.1016/S0022-2836(05)80360-2 2231712

[47] Untergasser A, Cutcutache I, Koressaar T, Ye J, Faircloth B, Remm M, et al Primer3—new capabilities and interfaces. Nucleic Acids Res. 2012;40: e115–e115. 10.1093/nar/gks596 22730293 PMC3424584

[48] Zhao S, Poulin L, Rodriguez-R LM, Serna NF, Liu S-Y, Wonni I, et al Development of a Variable Number of Tandem Repeats Typing scheme for the bacterial rice pathogen *Xanthomonas oryzae pv*. *oryzicola*. Phytopathology. 2012;102: 948–956. 10.1094/PHYTO-04-12-0078-R 22957820

[49] Yuan H, Qing LB, Zhi JD, Hua HL, Xia TX, Zhong ZJ. Identification of Variable-Number Tandem-Repeat (VNTR) sequences in *Acinetobacter pittii* and development of an optimized Multiple-Locus VNTR Analysis Typing scheme. Biomed Env Sci. 2015; 9. 10.3967/bes2015.119 26777905

[50] Monteil M, Durand B, Bouchouicha R, Petit E, Chomel B, Arvand M, et al Development of discriminatory multiple-locus variable number tandem repeat analysis for *Bartonella henselae*. Microbiology. 2007;153: 1141–1148. 10.1099/mic.0.2006/001164-0 17379723

[51] Kamvar ZN, Tabima JF, Grünwald NJ. *Poppr*: an R package for genetic analysis of populations with clonal, partially clonal, and/or sexual reproduction. PeerJ. 2014;2: e281 10.7717/peerj.281 24688859 PMC3961149

[52] Kamvar ZN, Brooks JC, Grünwald NJ. Novel R tools for analysis of genome-wide population genetic data with emphasis on clonality. Front Genet. 2015;6 10.3389/fgene.2015.00208 26113860 PMC4462096

[53] Hunter PR, Gaston MA. Numerical index of the discriminatory ability of typing systems: an application of Simpson’s index of diversity. J Clin Microbiol. 1988;26: 2465–2466. 10.1128/JCM.26.11.2465-2466.1988 3069867 PMC266921

[54] Excoffier L, Lischer HE. Arlequin suite ver 3.5: a new series of programs to perform population genetics analyses under Linux and Windows. Mol Ecol Resour. 2010;10: 564–567. 10.1111/j.1755-0998.2010.02847.x 21565059

[55] Kalinowski ST. hp-rare 1.0: a computer program for performing rarefaction on measures of allelic richness. Mol Ecol Notes. 2005;5: 187–189.

[56] Pelé J, Bécu J-M, Abdi H, Chabbert M. Bios2mds: an R package for comparing orthologous protein families by metric multidimensional scaling. BMC Bioinformatics. 2012;13: 133 10.1186/1471-2105-13-133 22702410 PMC3403911

[57] Borg I, Groenen PJ. Modern multidimensional scaling: theory and applications. Springer-Verlag New York; 2005.

[58] Francisco AP, Vaz C, Monteiro PT, Melo-Cristino J, Ramirez M, Carriço JA. PHYLOViZ: phylogenetic inference and data visualization for sequence based typing methods. BMC Bioinformatics. 2012;13: 87 10.1186/1471-2105-13-87 22568821 PMC3403920

[59] Abdurahman A, Parker ML, Kreuze J, Elphinstone J, Struik PC, Kiggundu A, et al Molecular epidemiology of *Ralstonia solanacearum* species complex strains causing bacterial wilt of potato in Uganda. Phytopathology. 2019; PHYTO-12-18-0476-R. 10.1094/PHYTO-12-18-0476-R 31272278

[60] Abdurahman A, Griffin D, Elphinstone J, Struik PC, Schulz S, Schulte-Geldermann E, et al Molecular characterization of *Ralstonia solanacearum* strains from Ethiopia and tracing potential source of bacterial wilt disease outbreak in seed potatoes. Plant Pathol. 2017;66: 826–834. 10.1111/ppa.12661

[61] Keim P, Van Ert MN, Pearson T, Vogler AJ, Huynh LY, Wagner DM. Anthrax molecular epidemiology and forensics: using the appropriate marker for different evolutionary scales. Infect Genet Evol. 2004;4: 205–213. 10.1016/j.meegid.2004.02.005 15450200

[62] Estoup A, Jarne P, Cornuet J-M. Homoplasy and mutation model at microsatellite loci and their consequences for population genetics analysis. Mol Ecol. 2002;11: 1591–1604. 10.1046/j.1365-294x.2002.01576.x 12207711

[63] Samad HM. Analyse de la filière tomate au lac Alaotra. Université d’Antananarivo; 2008.

[64] FERT. Etude de la filière légumes sur les Hautes Terres de Madagascar régions Analamanga, Itasy, Vakinankaratra, Amoron’i Mania: pomme de terre, tomate, oignon, carotte, haricot vert, et chou. 2012 p. 88.

[65] Liu Y, Wu D, Liu Q, Zhang S, Tang Y, Jiang G, et al The sequevar distribution of *Ralstonia solanacearum* in tobacco-growing zones of China is structured by elevation. Eur J Plant Pathol. 2017;147: 541–551. 10.1007/s10658-016-1023-6

[66] Rakotofiringa FMD. Caractérisation climatique des 22 chefs-lieux de région de Madagascar en utilisant les indices climatiques. Université d’Antananarivo; 2019.

[67] N’Guessan CA, Abo K, Fondio L, Chiroleu F, Lebeau A, Poussier S, et al So near and yet so far: the specific case of *Ralstonia solanacearum* populations from Côte d’Ivoire in Africa. Phytopathology. 2012;102: 733–740. 10.1094/PHYTO-11-11-0300 22533876

[68] Santiago TR, Lopes CA, Caetano-Anolles G, Mizubuti ESG. Genetic structure of *Ralstonia solanacearum* and *Ralstonia pseudosolanacearum* in Brazil. Plant Dis. 2019; PDIS-09-19-1929-RE. 10.1094/PDIS-09-19-1929-RE 31994983

[69] Deberdt P, Guyot J, Coranson-Beaudu R, Launay J, Noreskal M, Rivière P, et al Diversity of *Ralstonia solanacearum* in French Guiana expands knowledge of the “emerging ecotype”. Phytopathology. 2014;104: 586–596. 10.1094/PHYTO-09-13-0264-R 24283538

[70] Ramsubhag A, Lawrence D, Cassie D, Fraser R, Umaharan P, Prior P, et al Wide genetic diversity of *Ralstonia solanacearum* strains affecting tomato in Trinidad, West Indies: Tomato bacterial wilt strains in Trinidad. Plant Pathol. 2012;61: 844–857.

[71] Lin C-H, Tsai K-C, Prior P, Wang J-F. Phylogenetic relationships and population structure of *Ralstonia solanacearum* isolated from diverse origins in Taiwan. Plant Pathol. 2014;63: 1395–1403. 10.1111/ppa.12209

[72] Jiang G, Wei Z, Xu J, Chen H, Zhang Y, She X, et al Bacterial wilt in China: history, current status, and future perspectives. Front Plant Sci. 2017;8: 1549 10.3389/fpls.2017.01549 28955350 PMC5601990

[73] Carstensen GD, Venter SN, Wingfield MJ, Coutinho TA. Two *Ralstonia* species associated with bacterial wilt of *Eucalyptus*. Plant Pathol. 2017;66: 393–403. 10.1111/ppa.12577

[74] Pastou D, Chéron JJ, Cellier G, Guérin F, Poussier S. First report of *Ralstonia pseudosolanacearum* phylotype I causing bacterial wilt in New Caledonia. Plant Dis. 2019; PDIS-05-19-1068-PDN. 10.1094/PDIS-05-19-1068-PDN

[75] Chesneau T, Maignien G, Boyer C, Chéron J-J, Roux-Cuvelier M, Vanhuffel L, et al Sequevar diversity and virulence of *Ralstonia solanacearum* phylotype I on Mayotte Island (Indian Ocean). Front Plant Sci. 2018;8 10.3389/fpls.2017.02209 29354148 PMC5760537

[76] Milling A, Meng F, Denny TP, Allen C. Interactions with hosts at cool temperatures, not cold tolerance, explain the unique epidemiology of *Ralstonia solanacearum* race 3 biovar 2. Phytopathology. 2009;99: 1127–1134. 10.1094/PHYTO-99-10-1127 19740025

[77] Cellier G, Prior P. Deciphering phenotypic diversity of *Ralstonia solanacearum* strains pathogenic to potato. Phytopathology. 2010;100: 1250–1261. 10.1094/PHYTO-02-10-0059 20672871

[78] Tjou-Tam-Sin NNA, van de Bilt JLJ, Westenberg M, Gorkink-Smits PPMA, Landman NM, Bergsma-Vlami M. Assessing the pathogenic ability of *Ralstonia pseudosolanacearum* (*Ralstonia solanacearum* phylotype I) from ornamental *Rosa spp*. plants. Front Plant Sci. 2017;8: 1895 10.3389/fpls.2017.01895 29163615 PMC5673649

[79] Janse JD. Potato brown rot in Western Europe–history, present occurrence and some remarks on possible origin, epidemiology and control strategies. EPPO Bull. 1996;26: 679–695.

[80] Kim SH, Olson TN, Schaad NW, Moorman GW. *Ralstonia solanacearum* race 3, biovar 2, the causal agent of brown rot of potato, identified in geraniums in Pennsylvania, Delaware, and Connecticut. Plant Dis. 2003;87: 450–450. 10.1094/PDIS.2003.87.4.450C 30831858

[81] Blomme G, Dita M, Jacobsen KS, Pérez Vicente L, Molina A, Ocimati W, et al Bacterial diseases of bananas and enset: current state of knowledge and integrated approaches toward sustainable management. Front Plant Sci. 2017;8: 1290 10.3389/fpls.2017.01290 28785275 PMC5517453

[82] Bergsma-Vlami M, van de Bilt JLJ, Tjou-Tam-Sin NNA, Westenberg M, Meekes ETM, Teunissen HAS, et al Phylogenetic assignment of *Ralstonia pseudosolanacearum* (*Ralstonia solanacearum* phylotype I) isolated from *Rosa spp*. Plant Dis. 2018;102: 2258–2267. 10.1094/PDIS-09-17-1345-RE 30192708

[83] Yahiaoui N, Chéron JJ, Jeetah R, Benimadhu S, Félicité J, Cellier G, et al First report of *Ralstonia pseudosolanacearum* phylotype I causing bacterial wilt on Rodrigues Island, Indian Ocean. Plant Dis. 2016;100: 2522 10.1094/PDIS-06-16-0811-PDN

[84] Ramesh R, Gaitonde S, Achari G, Asolkar T, Singh NP, Carrere S, et al Genome sequencing of *Ralstonia solanacearum* biovar 3, phylotype I, strains Rs-09-161 and Rs-10-244, isolated from eggplant and chili in India. Genome Announc. 2014;2: e00323-14, 2/3/e00323-14. 10.1128/genomeA.00323-14 24874667 PMC4038872

[85] Kyaw HWW, Tsuchiya K, Matsumoto M, Iiyama K, Aye SS, Zaw M, et al Genetic diversity of *Ralstonia solanacearum* strains causing bacterial wilt of solanaceous crops in Myanmar. J Gen Plant Pathol. 2017;83: 216–225. 10.1007/s10327-017-0720-0

[86] Wicker E, Lefeuvre P, de Cambiaire J-C, Lemaire C, Poussier S, Prior P. Contrasting recombination patterns and demographic histories of the plant pathogen Ralstonia solanacearum inferred from MLSA. ISME J. 2012;6: 961–974. 10.1038/ismej.2011.160 22094345 PMC3329105

[87] Coupat B, Chaumeille-Dole F, Fall S, Prior P, Simonet P, Nesme X, et al Natural transformation in the *Ralstonia solanacearum* species complex: number and size of DNA that can be transferred. FEMS Microbiol Ecol. 2008;66: 14–24. 10.1111/j.1574-6941.2008.00552.x 18662313

[88] Pradhanang PM, Elphinstone JG, Fox RTV. Identification of crop and weed hosts of *Ralstonia solanacearum* biovar 2 in the hills of Nepal. Plant Pathol. 2000;49: 403–413. 10.1046/j.1365-3059.2000.00480.x

[89] Tusiime G, Adipala E, Opio F, Bhagsari AS. Weeds as latent hosts of Ralstonia solanacearum in highland Uganda: implications to development of an integrated control package for bacterial wilt. Elphinstone J. Bacterial Wilt Disease. Elphinstone J. Berlin, Heidelberg; 1998.

[90] Ciampi L, Sequeira L, French ER. Latent infection of potato tubers by *Pseudomonas solanacearum*. Am Potato J. 1980;57: 377–386. 10.1007/BF02854329

