# Supplementary figures and images for: Contrasting genetic diversity and structure among Malagasy Ralstonia pseudosolanacearum phylotype I populations inferred from an optimized Multilocus Variable Number of Tandem Repeat Analysis scheme
Source: PLoS One. 2020 Dec 8;15(12):e0242846. doi: 10.1371/journal.pone.0242846 (PMC7723262; doi:10.1371/journal.pone.0242846)

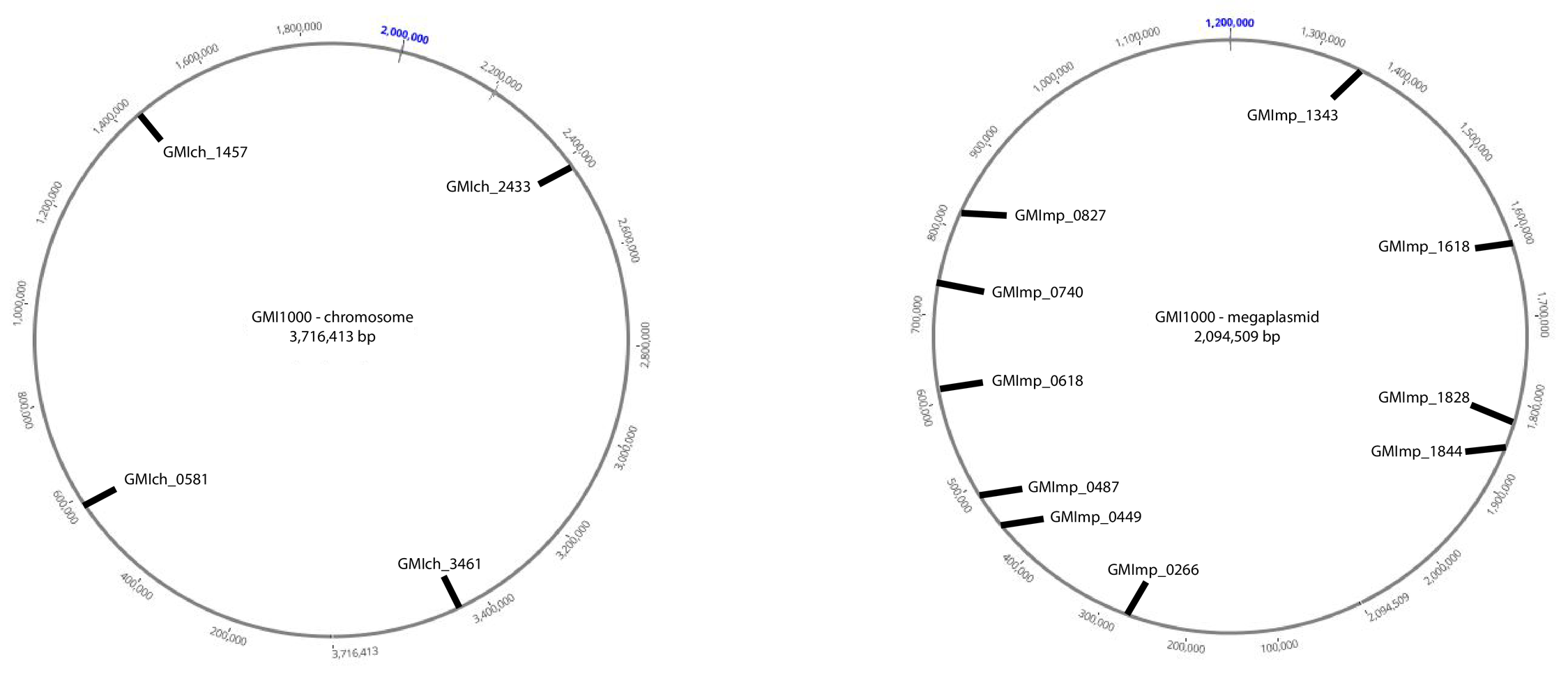

Supplement: S1 Fig — (TIF) [file pone.0242846.s008.tif]

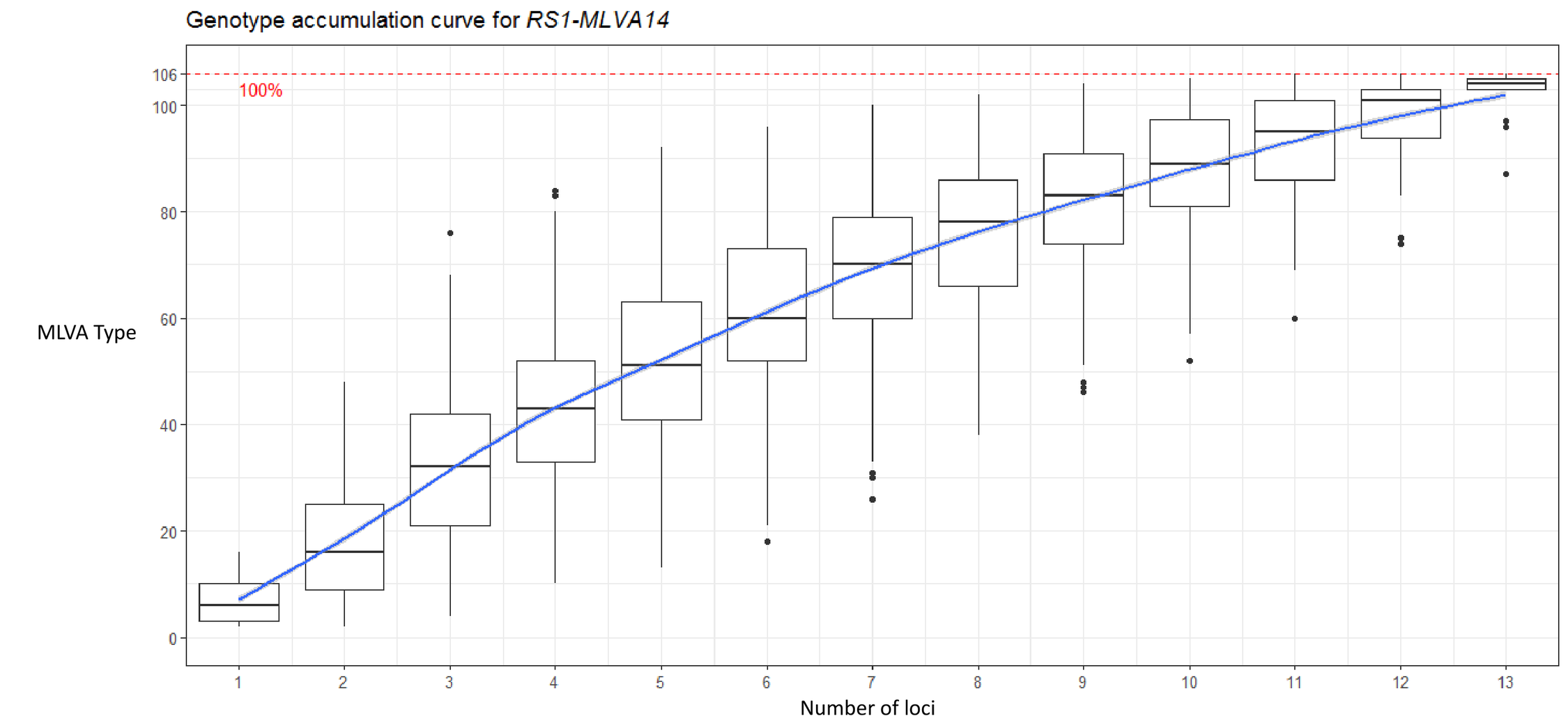

Supplement: S2 Fig — The horizontal axis represents the number of loci randomly sampled without replacement up to n-1 loci, the vertical axis shows the number of unique MLVA types observed (n = 107) in the data set. The level of 100% of unique MLVA types detected is indicated with a dotted red line. (TIF) [file pone.0242846.s009.tif]

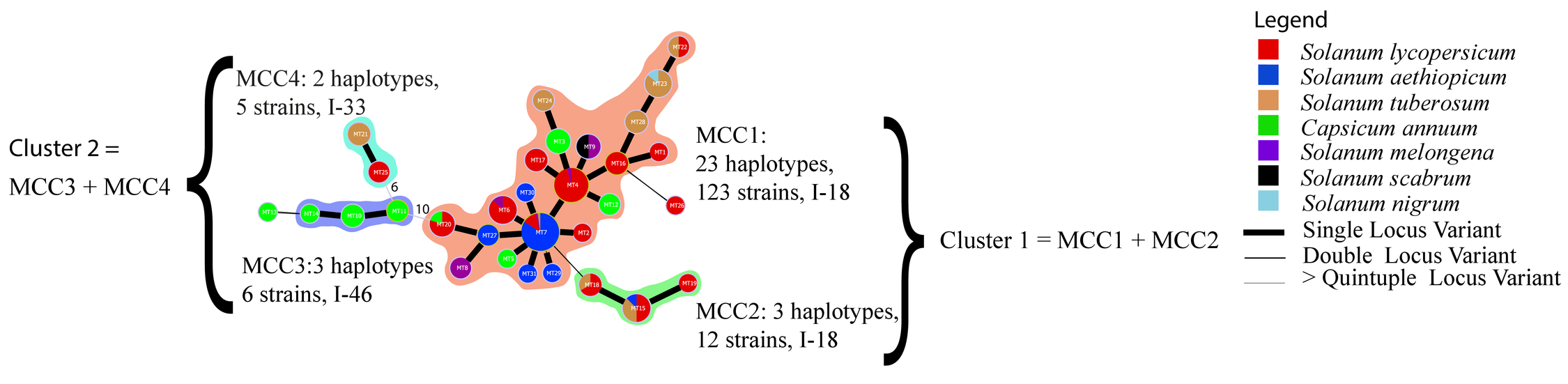

Supplement: S3 Fig — The haplotypes were identified by using goeBURST full MST in PHYLOVIZ. Each MLVA type (MT) is displayed as a circle, the size of which is proportional to the number of isolates represented. The different colours indicate the sample host. Branch thickness indicates the number of locus differences between the neighbouring haplotypes. MCC1, MCC2, MCC3, MCC4 represent the Malagasy Clonal Complexes 1, 2, 3 and 4. A clonal complex is composed of haplotypes that differ only by one VNTR locus. I-18, I-33 and I-46 represent the phylotype-sequevar of the isolates. (TIF) [file pone.0242846.s010.tif]
